# Interfacial Electric Effects on a Non-Isothermal Electroosmotic Flow in a Microcapillary Tube Filled by Two Immiscible Fluids

**DOI:** 10.3390/mi8080232

**Published:** 2017-07-27

**Authors:** Andrés Matías, Federico Méndez, Oscar Bautista

**Affiliations:** 1 Facultad de Ingeniería, Departamento de Termofluidos, Universidad Nacional Autónoma de México (UNAM), México City 04510, Mexico; mac_zog@hotmail.com (A.M.); fmendez@unam.mx (F.M.); 2 SEPI-ESIME Azcapotzalco, Instituto Politécnico Nacional (IPN), México City 02250, Mexico

**Keywords:** power-law fluid, electroosmotic flow, immiscible fluids, non-isothermal, microcapillary, Maxwell stress

## Abstract

In this work, a non-isothermal electroosmotic flow of two immiscible fluids within a uniform microcapillary is theoretically studied. It is considered that there is an annular layer of a non-Newtonian liquid, whose behavior follows the power-law model, adjacent to the inside wall of the capillary, which in turn surrounds an inner flow of a second conducting liquid that is driven by electroosmosis. The inner fluid flow exerts an interfacial force, dragging the annular fluid due to shear and Maxwell stresses at the interface between the two fluids. Because the Joule heating effect may be present in electroosmotic flow (EOF), temperature gradients can appear along the microcapillary, making the viscosity coefficients of both fluids and the electrical conductivity of the inner fluid temperature dependent. The above makes the variables of the flow field in both fluids, velocity, pressure, temperature and electric fields, coupled. An additional complexity of the mathematical model that describes the electroosmotic flow is the nonlinear character due to the rheological behavior of the surrounding fluid. Therefore, based on the lubrication theory approximation, the governing equations are nondimensionalized and simplified, and an asymptotic solution is determined using a regular perturbation technique by considering that the perturbation parameter is associated with changes in the viscosity by temperature effects. The principal results showed that the parameters that notably influence the flow field are the power-law index, an electrokinetic parameter (the ratio between the radius of the microchannel and the Debye length) and the competition between the consistency index of the non-Newtonian fluid and the viscosity of the conducting fluid. Additionally, the heat that is dissipated trough the external surface of the microchannel and the sensitivity of the viscosity to temperature changes play important roles, which modify the flow field.

## 1. Introduction

Fluid transport is an essential task in microfluidic devices, where electroosmotic pumping (EOP) can be used [1,2] as an effective tool for displacing fluids and suspended particles in microchannels. EOP refers to the motion of an electrolyte solution relative to a stationary charged surface when an electric potential is applied [3]. However, electroosmosis cannot be directly used to drive non-conducting fluids. For this purpose, Brask et al. [4] proposed an electroosmotic pump that relies on two-liquid viscous drag to pump non-conducting liquids.

The pumping of non-conducting fluids has been widely studied by several researchers. Gao et al. [5] presented a numerical analysis of the electroosmotic flow (EOF) in a microchannel for two stratified immiscible liquids with low diffusivity: a high electroosmotic (EO) mobility liquid at the bottom section and a low EO liquid at the upper section of the channel. The main results indicated that the interface between the two fluids can be controlled by electroosmotic effects. Gao et al. [6] analyzed the transient aspects of two-liquid EOF, in which a low EO mobility liquid is delivered by the interfacial viscous force of a high EO mobility liquid driven by electroosmosis. These authors included the effects of the dynamic viscosity ratio, interface potential, kinematic viscosity ratio and the parameter of the electrical double layer (EDL) to characterize the flow. Choi et al. [7] studied a two-fluid EOF in a microchannel by considering full hydrodynamic and electric interactions on the interface, and both fluids were assumed to be Newtonian fluids. These authors demonstrated that interfacial electrostatic effects induce a flow reversal. In the same context, Movahed et al. [8] conducted a numerical simulation of the EOF in a column of an aqueous solution surrounded by an immiscible liquid. The effects of the EDL and surface charge were considered for the boundary conditions at the interface of the two fluids. In addition to these studies, a time-dependent model of mixed electroosmotic/pressure-driven flow of three immiscible fluids in a rectangular microchannel was developed by Haiwang et al. [9], who studied a physical model where a non-conducting fluid is driven by a pressure gradient and interfacial viscous forces of two conducting liquids, which are driven by electroosmotic forces. The aforementioned investigations focus on studying the electroosmotic-driven flow of Newtonian fluids; however, in many applications of EOFs, the fluids transported through microchannels are non-Newtonian fluids. In this direction, Huang et al. [10] analyzed a physical electroosmotic model based on two immiscible layers with one layer of a conducting non-Newtonian fluid, whose rheological behavior is described by a power law. The results demonstrated that the fluid consistency coefficient and flow behavior index of the fluid influence notably impact the shape of the velocity profile and the volume flow rate. Afonso et al. [11] developed an analytical model for a two-fluid EOF of stratified fluids with Newtonian or viscoelastic rheological behavior. The effects of fluid rheology, shear viscosity ratio and interfacial zeta potential were analyzed, revealing that an enhancement of the flow rate is observed as the shear-thinning effects are increased. Liu et al. [12] analytically solved the EOP of nonconducting liquids and biofluids in a circular microchannel, where two models were proposed: (1) the conducting layer is a Newtonian fluid, and the inner layer is a conducting Casson fluid; and (2) both of the layers are Newtonian fluids. Analytical solutions of the electric potential distribution, velocity profile, flow rate and electric current were obtained.

As previously demonstrated, Joule heating is inevitable when an electric field is applied across a conducting medium, which imposes limitations on the performance of electrokinetic microfluidic devices [13] or can significantly modify the flow and electric fields [14,15,16]. In this direction, although there are several works investigating the hydrodynamic aspects of the EOF of immiscible fluids, thermal analyses related to this topic are scarce. For instance, Garai and Chakraborty [17] performed a theoretical analysis of the heat transfer in a combined electroosmotic and pressure-driven flow of two immiscible Newtonian liquid layers in a microchannel, where the fully-developed flow condition was assumed. The velocity and temperature profiles in the two fluids, together with the Nusselt number, were obtained.

Although thermal analyses of EOF with immiscible fluids have been conducted, none of these analyses consider the variation of the physical properties due to temperature, which may change considerably due to the relatively high external electric fields used in EOF. Additionally, the simultaneous effects of viscous and Maxwell stresses with non-isothermal conditions have been considered. In this sense, few works [8,12] have studied the electroosmotic flow of immiscible fluids in cylindrical coordinates. In the case of [8], the analysis considered two immiscible Newtonian fluids in a microcapillary, under isothermal conditions. The solution was conducted by using the COMSOL software. In [12], the analytical solution for a coaxial two-phase electroosmotic flow in a circular microchannel was studied and solved in analytical fashion, but isothermal conditions were assumed. In this last reference, non-Newtonian fluids were considered; however, for such cases, viscous stresses at the interface between both fluids were included, and Maxwell stresses were neglected. Only in the case of Newtonian fluids, viscous and Maxwell stresses were considered in a simultaneous manner. Another important aspect to consider from the present work is that we found an approximate solution, based on a regular perturbation technique [18], of the the non-linear and coupled partial differential equations (mass, momentum, energy, charge and electric field) that describe this EOF. In addition, the boundary conditions at the interface between both fluids take into account viscous and Maxwell stresses, continuity of velocity, temperature and heat flux, which couple the field variables between the Newtonian and non-Newtonian fluids.

## 2. Theoretical Model

**Figure 1:**
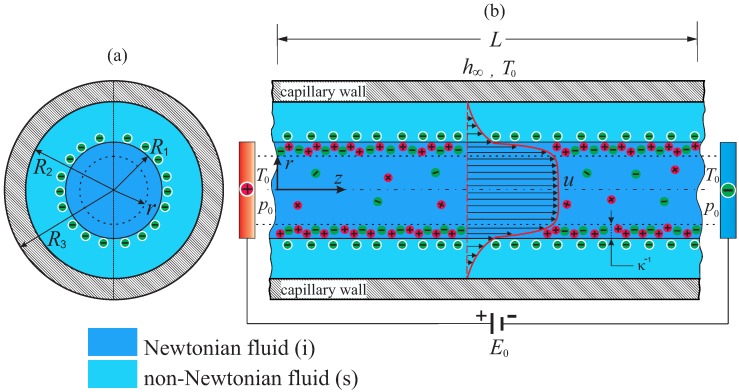
(Color online) Schematic of the electroosmotic flow of two immiscible fluids in a microcapillary: (**a**) cross-sectional view and (**b**) side view, depicting both fluids in distinct colors.

Figure 1 presents the scheme of the physical model analyzed in this work. A microcapillary, with length *L* that is considerably greater than its inner radius $R_{2}$, is filled with two immiscible fluids with an annular arrangement. A 2D cylindrical coordinate system (r,z) is adopted, and the origin is placed at the left end of the capillary. The column of the inner fluid (denoted *i*), whose radius is $R_{1}$, is a symmetric electrolyte with Newtonian behavior, and the surrounding non-conducting fluid (denoted *s*) obeys the power-law rheological model. A thin EDL (with thickness given by the Debye length, $\kappa^{-1}$) is formed at the liquid-liquid interface on the conducting fluid side. The inner fluid flow is driven by an electroosmotic force due to an external electric field of intensity $E_{0}$ in the axial direction, which is given by $E_{0}=\phi_{0}/L$, where $\phi_{0}$ is the electric potential imposed at the entrance of the capillary in z=0. The thickness of the capillary wall is denoted by $t_{w}=R_{3}-R_{2}$, where $R_{3}$ is the external radius of the capillary. The capillary ends are supported by two reservoirs that are found at temperature $T_{0}$ and pressure $p_{0}$. Additionally, the outer surface of the capillary is in contact with the surroundings; for simplicity, its temperature is also $T_{0}$, and its convective heat transfer coefficient is $h_{\infty }$.

The following assumptions are also made: (i) the viscosity and electrical conductivity of the electrolyte solution, as well as the consistency index of the non-Newtonian fluid are functions of temperature, whereas the thermal conductivities of both fluids are constant because this property is considerably less sensitive to temperature variations [19,20]; (ii) the radius $R_{1}$ of the capillary is considerably greater than the Debye length, $\kappa^{-1}$; and (iii) the boundary between the two fluids is well defined and stable, i.e., the liquid film thickness $t=R_{2}-R_{1}$ is constant along the microcapillary. In this context, the very small pressure difference that arose from surface tension and curvature was ignored [21]. This is a restrictive assumption; however, we assume that the position of the interface remains unaltered because the capillary number is very small, i.e., $Ca=\epsilon E_{0}\psi_{c}/\gamma_{T}\ll 1$ [22]; for instance, typical values of the physical parameters used in this study take the following values: the dielectric permittivity is $\epsilon ∼7\times 10^{-10}$ C·$V^{-1}$·$m^{-1}$, the external electric field $E_{0}∼10^{4}$ V·$m^{-1}$, the thermal voltage or characteristic electric potential in the EDL, defined later, $\psi_{c}\leq 25$ m·V, and the surface tension between both fluids $\gamma_{T}∼10^{-3}$ N·$m^{-1}$. With these values, the capillary number is estimated as $Ca∼10^{-4}$. Of course, for higher values of the surface tension, such as $\gamma_{T}∼10^{-2}$ N·$m^{-1}$, the capillary number is decreased, i.e., $Ca∼10^{-5}$. (iv) The electric double layer thickness is assumed to be uniform along the interface between both fluids; this assumption should be relaxed in a future work. We do consider that the electric double layer thickness is not affected by temperature gradients. In this sense, thermodiffusion, ionic diffusivities and thermodiffusion coefficients are not taken into account in this analysis. For a further discussion about these issues, see [23,24,25,26,27] and the references cited therein. Finally, (v) viscous dissipation is neglected in comparison with the Joule heating effect [28,29].

Considering the aforementioned together with the lubrication approximation for this non-isothermal EOF [30], the governing equations can be described as shown below.

### 2.1. For the Conducting Fluid

The governing equations that describe the hydrodynamics, temperature and electric fields in the conducting fluid are given by the continuity, momentum, energy and charge conservation equations:

(1) $$\frac{1}{r}\frac{\partial \left(rv_{i}\right)}{\partial r}+\frac{\partial u_{i}}{\partial z}=0,$$

(2) $$\frac{1}{r}\frac{\partial }{\partial r}\left[\mu \left(T_{i}\right)r\left(\frac{\partial u_{i}}{\partial r}\right)\right]+\rho_{e}E_{z}-\frac{dp_{i}}{dz}=0,$$

(3) $$\rho_{i}C_{p,i}\left(u_{i}\frac{\partial T_{i}}{\partial z}\right)=k_{i}\left[\frac{\partial^{2}T_{i}}{\partial z^{2}}+\frac{1}{r}\frac{\partial }{\partial r}\left(r\frac{\partial T_{i}}{\partial r}\right)\right]+\sigma \left(T_{i}\right)E_{z}^{2}$$

and:

(4) $$\frac{d}{dz}\left[\sigma \left(T_{i}\right)\frac{d\phi_{i}}{dz}\right]=0.$$

In Equations (1)–(4), $v_{i}$ and $u_{i}$ are the velocity components in the *r* and *z* directions, respectively; $p_{i}$ and $T_{i}$ are the pressure and temperature fields, respectively; and the electric field along the capillary is defined as $E_{z}=-d\phi_{i}/dz$, where $\phi_{i}$ is the external electric potential. The electrical conductivity and viscosity of the conducting fluid are temperature dependent and are defined as $\sigma \left(T_{i}\right)=\sigma_{0}\left[1+B_{\sigma }\left(T_{i}-T_{0}\right)\right]$ [30,31] and $\mu \left(T_{i}\right)=\mu_{0}\exp\left(B_{\mu }/T_{i}\right)$ [30,32], respectively. Here, $\sigma_{0}$ and $\mu_{0}$ are the electrical conductivity and viscosity evaluated at a reference temperature $T_{0}$, and $B_{\sigma }$ and $B_{\mu }$ are constants that measure the sensitivity of the electrical conductivity and viscosity to temperature. $k_{i}$, $\rho_{i}$ and $C_{p,i}$ are the thermal conductivity, the mass density and the specific heat, respectively.

In Equation (2), the charge density, $\rho_{e}$, is obtained from the Poisson–Boltzmann equation. For very long microchannels and assuming that the zeta potential at the liquid-liquid interface is small, i.e., ζ≪25 mV, such that the Debye–Hückel approximation can be used, this equation can be written as:

(5) $$\frac{1}{r}\frac{d}{dr}\left(r\frac{d\psi }{dr}\right)=-\frac{\rho_{e}}{\epsilon }.$$

By considering that $\rho_{e}=-\epsilon \kappa^{2}\psi$ and that the boundary conditions of Equation (5) are $\psi \left(r=R_{1}\right)=\zeta$ and dψ/dr=0 at r=0, the charge density is obtained as $\rho_{e}=-\epsilon \kappa^{2}\zeta I_{0}\left(\kappa r\right)/I_{0}\left(\kappa R_{1}\right)$ [31]. Here, ψ is the electric potential within the Debye length; ϵ denotes the dielectric permittivity of the conducting fluid; $I_{0}$ is the zeroth-order modified Bessel function [33]; and κ is the inverse Debye screening thickness, defined as $\kappa ={\left(2e^{2}z^{2}n_{\infty }/\epsilon k_{B}T_{0}\right)}^{-1/2}$, where *e*, $n_{\infty }$, *z*, $k_{B}$ and $T_{0}$ are the magnitude of the elementary charge on an electron, the bulk concentration of ions, the valence, the Boltzmann constant and an absolute reference temperature $T_{0}$, respectively.

### 2.2. For the Non-Conducting Fluid

The governing equations in the region of the non-conducting liquid are the continuity, momentum and the energy equations, which are given by:

(6) $$\frac{1}{r}\frac{\partial \left(rv_{s}\right)}{\partial r}+\frac{\partial u_{s}}{\partial z}=0,$$

(7) $$-\frac{1}{r}\frac{\partial }{\partial r}\left[m\left(T_{s}\right)r{\left(-\frac{\partial u_{s}}{\partial r}\right)}^{n}\right]-\frac{dp_{s}}{dz}=0,$$

(8) $$\rho_{s}C_{p,s}\left(u_{s}\frac{\partial T_{s}}{\partial z}\right)=k_{s}\left[\frac{\partial^{2}T_{s}}{\partial z}+\frac{1}{r}\frac{\partial }{\partial r}\left(r\frac{\partial T_{s}}{\partial r}\right)\right].$$

Here, $v_{s}$, $u_{s}$, $p_{s}$ and $T_{s}$ are the velocity components in the *r* and *z* directions, the pressure field and the temperature, respectively. $m\left(T_{s}\right)=m_{0}\exp\left[-a\left(T_{s}-T_{0}\right)\right]$ represents the consistency index for the non-conducting fluid [34], with $m_{0}$ denoting the consistency index evaluated at the reference temperature $T_{0}$, and *a* is a parameter related with the sensitivity of the consistency index to temperature variations. $k_{s}$, $\rho_{s}$ and $C_{p,s}$ are the thermal conductivity, the mass density and the specific heat of the non-conducting fluid, respectively, and *n* is the flow behavior index. Note that in the energy equations, Equations (3) and (8), the convective terms in the radial direction have been neglected, as proven by Sánchez et al. [16].

#### Boundary Conditions

The boundary conditions of the governing Equations (1)–(8) are as follows:

At the capillary centerline, r=0, the symmetry boundary conditions for velocity and temperature are applied:

(9) $$v_{i}=0,\;\frac{\partial u_{i}}{\partial r}=0,\;\frac{\partial T_{i}}{\partial r}=0;$$

The matching conditions at the interface between both fluids ($r=R_{1}$) are:

(10) $$u_{i}=u_{s},\;v_{i}=v_{s}=0,$$

(11) $$\tau_{rz,i}-\tau_{rz,s}=\sigma_{s}E_{z},$$

(12) $$T_{i}=T_{s},\;k_{s}\frac{\partial T_{s}}{\partial r}=k_{i}\frac{\partial T_{i}}{\partial r}.$$

In Equation (10), the first and second conditions are the continuity and the impermeability of the velocity between both fluids, respectively. Equation (11) represents the total stress balance, which includes shear and Maxwell stresses [5]. In Equation (11), $\tau_{rz,i}=\mu \left(T_{i}\right)\left(\partial u_{i}/\partial r\right)$ and $\tau_{rz,s}=m\left(T_{s}\right){\left(-\partial u_{s}/\partial r\right)}^{n}$. Additionally, $\sigma_{s}=-\epsilon \kappa \zeta I_{1}\left(\kappa R_{1}\right)/I_{0}\left(\kappa R_{1}\right)$ is the surface charge density at the interface [31], and $I_{0}$ and $I_{1}$ are the zeroth- and first-order modified Bessel functions [33], respectively. Furthermore, the continuity of temperatures and heat flux are represented by Equation (12), which defines a conjugate heat transfer problem between the two fluids.

At the inner surface of the capillary ($r=R_{2}$), the boundary conditions are:

(13) $$u_{s}=v_{s}=0,\;\frac{\partial T_{s}}{\partial r}=-\frac{h_{eq}}{k_{s}}\left(T_{s}-T_{0}\right).$$

The first two boundary conditions in Equation (13) are the no-slip and impermeability conditions at the inner surface of the capillary; the latter represents the convective cooling from the capillary outer surface, where $h_{eq}$ is the equivalent heat transfer coefficient given by:

(14) $$h_{eq}=R_{2}^{-1}{\left[\frac{1}{k_{w}}\ln\left(\frac{R_{3}}{R_{2}}\right)+\frac{1}{h_{\infty }R_{3}}\right]}^{-1}.$$

Here, $k_{w}$ is the thermal conductivity of the microchannel wall. At both ends of the capillary (z=0,L):

(15) $$p_{i}=p_{s}=p_{0},\;T_{i}=T_{s}=T_{0},$$

and:

(16) $$\phi_{i}\left(z=0\right)=\phi_{0},\;\phi_{i}\left(z=L\right)=0.$$

Finally, both ends of the capillary are at the same pressure $p_{0}$ and the same temperature $T_{0}$, as represented by Equation (15); this latter condition reflects the cooling from the capillary ends, as used by Xuan et al. [14]. The applied electric potential at the capillary inlet is represented by $\phi =\phi_{0}$, and ϕ=0 denotes that the capillary outlet is grounded.

### 2.3. Dimensionless Equations

To analyze this EOF and because there are many physical parameters involved in the analysis, we first nondimensionalize the governing Equations (1)–(8), together with the boundary conditions (9)–(16), by introducing the following dimensionless variables:

(17) $$\begin{array}{c} Z=\frac{r-R_{1}}{t},\;\chi =\frac{x}{L}\;\eta =\frac{r}{R_{1}},\;{\bar{u}}_{i}=\frac{u_{i}}{u_{c}},\;{\bar{u}}_{s}=\frac{u_{s}}{u_{c}},\\ {\bar{v}}_{i}=\frac{v_{i}L}{R_{1}u_{c}},\;{\bar{v}}_{s}=\frac{v_{s}L}{tu_{c}},\;{\bar{p}}_{i}=\frac{p_{i}-p_{0}}{\Delta p_{c}},\;\;{\bar{p}}_{s}=\frac{p_{s}-p_{0}}{\Delta p_{c}},\\ \theta_{i}=\frac{T_{i,s}-T_{0}}{\Delta T_{c}},\;\theta_{s}=\frac{T_{s}-T_{0}}{\Delta T_{c}},\;\bar{\phi }=\frac{\phi }{\phi_{0}}. \end{array}$$

Here, $u_{c}=\epsilon E_{0}\psi_{c}/\mu_{0}$ represents the Helmholtz–Smoluchowski velocity and is chosen to be the characteristic velocity for both fluids, and it is evaluated at the reference temperature $T_{0}$. $\psi_{c}=k_{B}T/ze$ denotes the thermal voltage. $\Delta T_{c}=\sigma_{0}E_{0}^{2}LR_{1}/k_{i}$ and $\Delta p_{c}=\mu_{0}u_{c}L/R_{1}^{2}$ represent the characteristic temperature increment and characteristic pressure drop in the system, respectively. Therefore, the dimensionless versions of the governing equations, Equations (1)–(4) and Equations (6)–(8), are as follows:

For the conducting fluid,

(18) $$\frac{1}{\eta }\frac{\partial }{\partial \eta }\left(\eta {\bar{v}}_{i}\right)+\frac{\partial {\bar{u}}_{i}}{\partial \chi }=0,$$

(19) $$\frac{1}{\eta }\frac{\partial }{\partial \eta }\left[\left(1-\gamma_{\mu }\;\theta_{i}\right)\eta \frac{\partial {\bar{u}}_{i}}{\partial \eta }\right]-{\bar{\kappa }}^{2}\frac{I_{0}\left(\bar{\kappa }\eta \right)}{I_{0}\left(\bar{\kappa }\right)}\frac{d{\bar{\phi }}_{i}}{d\chi }-\frac{d{\bar{p}}_{i}}{d\chi }=0$$

(20) $$\frac{\partial^{2}\theta_{i}}{\partial \chi^{2}}-\frac{Pe_{i}{\bar{u}}_{i}}{\beta_{i}}\frac{\partial \theta_{i}}{\partial \chi }+\frac{1}{\beta_{i}^{2}}\frac{1}{\eta }\frac{\partial }{\partial \eta }\left(\eta \frac{\partial \theta_{i}}{\partial \eta }\right)+\left[1+\gamma_{\sigma }\theta_{i}\right]{\left(\frac{d{\bar{\phi }}_{i}}{d\chi }\right)}^{2}=0,$$

(21) $$\frac{d}{d\chi }\left[\left(1+\gamma_{\sigma }\theta_{i}\right)\frac{d{\bar{\phi }}_{i}}{d\chi }\right]=0;$$

and for the non-conducting fluid,

(22) $$\frac{1}{\left(1+\xi Z\right)}\frac{\partial }{\partial Z}\left[\left(1+\xi Z\right){\bar{v}}_{s}\right]+\frac{\partial {\bar{u}}_{s}}{\partial \chi }=0,$$

(23) $$-\frac{\partial }{\partial Z}\left[\left(1+\xi Z\right)\left(1-\gamma_{a}\theta_{s}\right){\left(-\frac{\partial {\bar{u}}_{s}}{\partial Z}\right)}^{n}\right]-\Lambda \frac{d{\bar{p}}_{s}}{d\chi }\left(1+\xi Z\right)=0,$$

(24) $$\frac{\partial^{2}\theta_{s}}{\partial \chi^{2}}-\frac{Pe_{s}{\bar{u}}_{s}}{\beta_{s}}\frac{\partial \theta_{s}}{\partial \chi }+\frac{1}{\beta_{s}^{2}}\frac{1}{\left(1+\xi Z\right)}\frac{\partial }{\partial Z}\left[\left(1+\xi Z\right)\frac{\partial \theta_{s}}{\partial Z}\right]=0.$$

In Equations (18)–(24), $\bar{\kappa }=\kappa R_{1}$, $\gamma_{\mu }=B_{\mu }▵T_{c}/T_{0}^{2}$, $\gamma_{\sigma }=B_{\sigma }▵T_{c}$ and $\gamma_{a}=a▵T_{c}$. The other dimensionless parameters are defined as $\Lambda =t^{n+1}u_{c}^{1-n}/\mu_{r}R_{1}^{2}$, $\beta_{i}=R_{1}/L$, $\beta_{s}=t/L$ and $\xi =t/R_{1}$. $Pe_{i}=u_{c}R_{1}/\alpha_{i}$ and $Pe_{s}=u_{c}t/\alpha_{s}$ are the Péclet numbers for the inner and surrounding fluids, respectively, with $\alpha_{i,s}=k_{i,s}/\rho_{i,s}C_{p,i,s}$.

The dimensionless boundary conditions, corresponding to Equations (18)–(24), are as follows:

At the centerline of the capillary (η=0):

(25) $${\bar{v}}_{i}=0,\;\frac{\partial {\bar{u}}_{i}}{\partial \eta }=0,\;\frac{\partial \theta_{i}}{\partial \eta }=0.$$

At the interface between both fluids:

(26) $${\bar{u}}_{i}{|}_{\eta =1}={\bar{u}}_{s}{|}_{Z=0},\;{\bar{v}}_{i}{|}_{\eta =1}={\bar{v}}_{s}{|}_{Z=0}=0,$$

(27) $$\begin{array}{c} -\left(1-\gamma_{\mu }\theta_{i}\right)\frac{\partial {\bar{u}}_{i}}{\partial \eta }{|}_{\eta =1}-\alpha \left(1-\gamma_{a}\theta_{s}\right){\left(-\frac{\partial {\bar{u}}_{s}}{\partial Z}\right)}^{n}{|}_{Z=0}=\;\bar{\kappa }\frac{I_{1}\left(\bar{\kappa }\right)}{I_{0}\left(\bar{\kappa }\right)}\frac{d{\bar{\phi }}_{i}}{d\chi }, \end{array}$$

(28) $$\theta_{i}{|}_{\eta =1}=\theta_{s}{|}_{Z=0},\;\bar{\alpha }\frac{\partial \theta_{i}}{\partial \eta }{|}_{\eta =1}=\frac{\partial \theta_{s}}{\partial Z}{|}_{Z=0}.$$

Here, $\alpha =\mu_{r}R_{1}u_{c}^{n-1}/t^{n}$, with $\mu_{r}=m_{0}/\mu_{0}$. $\bar{\alpha }=k_{r}\xi$ denotes a conjugate heat transfer parameter, where $k_{r}=k_{i}/k_{s}$.

At the inner surface of the capillary (Z=1):

(29) $${\bar{u}}_{s}={\bar{v}}_{s}=0,\;\frac{\partial \theta_{s}}{\partial Z}=k_{T}\theta_{s},$$

where $k_{T}=h_{eq}t/k_{s}$ represents an equivalent Biot number.

At both ends of the capillary (χ=0,1):

(30) $${\bar{p}}_{i,s}=0,\;\theta_{i,s}=0$$

and:

(31) $${\bar{\phi }}_{i}\left(\chi =0\right)=1\;\mathrm{and}\;{\bar{\phi }}_{i}\left(\chi =1\right)=0.$$

#### Simplified Energy Equations

The energy equations for the inner and surrounding fluids, which are given by Equations (20) and (24), respectively, can be simplified based on the following discussion. In typical EOFs, the temperature variation over *r* is considerably smaller than that in the axial direction [30]; therefore, to a first approximation, the temperature for both fluids is only a function of the coordinate *z*. Thus, in dimensionless form, we have $\theta_{i,s}\approx \theta_{i,s}\left(\chi \right)$. Hence, it is possible to replace the local temperature in both fluids for the cross-sectional average temperature. Therefore, averaging each term in the radial direction of the energy equations for the inner and surrounding fluids and using the boundary conditions that define the conjugate heat transfer problem, namely, the second boundary conditions of Equations (28) and (29), yield the following set of equations:

(32) $$\frac{d^{2}\theta_{i}}{d\chi^{2}}-\frac{Pe_{i}\langle {\bar{u}}_{i}\rangle }{\beta_{i}}\frac{d\theta_{i}}{d\chi }+\frac{2}{\beta_{i}^{2}}{\left.\frac{\partial \theta_{i}}{\partial \eta }\right|}_{\eta =1}+\frac{1}{\beta_{i}}{\left(\frac{d{\bar{\phi }}_{i}}{d\chi }\right)}^{2}=0$$

and:

(33) $$\frac{d^{2}\theta_{s}}{d\chi^{2}}-\frac{Pe_{s}\langle {\bar{u}}_{s}\rangle }{\beta_{s}}\frac{d\theta_{s}}{d\chi }-\frac{2}{\beta_{s}^{2}\left(1+R_{r}\right)}\left[\left(1+\xi \right)k_{T}\theta_{s}+\bar{\alpha }{\left.\frac{\partial \theta_{i}}{\partial \eta }\right|}_{\eta =1}\right]=0,$$

respectively. Here, $R_{r}=R_{2}/R_{1}$, and ${\left(\partial \theta_{i}/\partial \eta \right)}_{\eta =1}$ is the unknown dimensionless temperature gradient at the interface between both fluids, which will be determined in Appendix A. $\langle {\bar{u}}_{i}\rangle$ and $\langle {\bar{u}}_{s}\rangle$ represent average velocities, which are defined as:

(34) $$\langle {\bar{u}}_{i}\left(\chi \right)\rangle =2\int_{0}^{1}{\bar{u}}_{i}\eta d\eta$$

and:

(35) $$\langle {\bar{u}}_{s}\left(\chi \right)\rangle =\frac{2}{1+R_{r}}\int_{0}^{1}{\bar{u}}_{s}\left(1+\xi Z\right)dZ.$$

## 3. Asymptotic Solution in the Limit of $\gamma_{\mu }\ll 1$

To solve the coupled system of the governing equations formulated in the previous section, we use a perturbation technique [18]. The governing Equations (18)–(31) depend on several small dimensionless parameters, such as $\gamma_{\mu }$, $\gamma_{\sigma }$ and $\gamma_{a}$. All of these parameters are defined in terms of the characteristic temperature increment $\Delta T_{c}$, which allows these parameters to be expressed in terms of a single parameter. Under this condition, we can write that $\gamma_{\sigma }=\Gamma_{\sigma }\gamma_{\mu }$ and $\gamma_{a}=\Gamma_{a}\gamma_{\mu }$, with $\Gamma_{\sigma }=B_{\sigma }T_{0}^{2}/B_{\mu }$ and $\Gamma_{a}=aT_{0}^{2}/B_{\mu }$. Therefore, the governing equations can be written in terms of the dimensionless parameter $\gamma_{\mu }$. From a physical perspective, this parameter measures the sensitivity of viscosity μ to changes in temperature. By considering the typical values of geometrical and physical properties used in EOF, such as $B_{\mu }=1713$ K, $E_{0}∼10^{4}$ V·$m^{-1}$, $k_{i}=0.609$ W·$m^{-1}$·$K^{-1}$, $L∼10^{-2}$ m [14], $R_{1}=50$ μm [8] and $T_{0}=298$ K, we estimate that $\gamma_{\mu }∼10^{-3}\ll 1,$ and therefore, we can consider this to be the perturbation parameter $\gamma_{\mu }$. Thus, we propose a regular expansion for each dependent variable (say, *X*) in the form:

(36) $$X=X^{\left(0\right)}+\gamma_{\mu }X^{\left(1\right)}+O\left(\gamma^{2}\right),$$

where $X=\theta_{i},\theta_{s},{\bar{u}}_{i},{\bar{u}}_{s},{\bar{v}}_{i},{\bar{v}}_{s},{\bar{p}}_{i},{\bar{p}}_{s},{\bar{\phi }}_{i}$. Substituting the expansions (36) into Equations (18)–(24), as well as into the boundary conditions (25)–(31) and collecting terms of the same order, we obtain the following set of equations.

### 3.1. Leading-Order Solution

Note that at this order, the solution corresponds to the case of constant physical properties, where the flow is strictly unidirectional, and the component of the radial velocity of the fluid does not exist. Thus, the leading-order equations are defined as:

(37) $$\frac{1}{\eta }\frac{\partial }{\partial \eta }\left[\eta \frac{\partial {\bar{u}}_{i}^{\left(0\right)}}{\partial \eta }\right]-{\bar{\kappa }}^{2}\frac{I_{0}\left(\bar{\kappa }\eta \right)}{I_{0}\left(\bar{\kappa }\right)}\frac{d{\bar{\phi }}_{i}^{\left(0\right)}}{d\chi }=0,$$

(38) $$-\frac{\partial }{\partial Z}\left[\left(1+\xi Z\right){\left(-\frac{\partial {\bar{u}}_{s}^{\left(0\right)}}{\partial Z}\right)}^{n}\right]=0,$$

(39) $$\frac{d^{2}\theta_{i}^{\left(0\right)}}{d\chi^{2}}-\frac{Pe_{i}\langle {\bar{u}}_{i}^{\left(0\right)}\rangle }{\beta_{i}}\frac{d\theta_{i}^{\left(0\right)}}{d\chi }+\frac{2}{\beta_{i}^{2}}{\left.\frac{\partial \theta_{i}^{\left(0\right)}}{\partial \eta }\right|}_{\eta =1}+\frac{1}{\beta_{i}}{\left(\frac{d{\bar{\phi }}_{i}^{\left(0\right)}}{d\chi }\right)}^{2}=0,$$

(40) $$\frac{d^{2}\theta_{s}^{\left(0\right)}}{d\chi^{2}}-\frac{Pe_{s}\langle {\bar{u}}_{s}^{\left(0\right)}\rangle }{\beta_{s}}\frac{d\theta_{s}^{\left(0\right)}}{d\chi }-\frac{2}{\beta_{s}^{2}\left(1+R_{r}\right)}\left[\left(1+\xi \right)k_{T}\theta_{s}^{\left(0\right)}+\bar{\alpha }{\left.\frac{\partial \theta_{i}^{\left(0\right)}}{\partial \eta }\right|}_{\eta =1}\right]=0,$$

and:

(41) $$\frac{d^{2}{\bar{\phi }}_{i}^{\left(0\right)}}{d\chi^{2}}=0.$$

The boundary conditions are given by:

(42) $$\frac{\partial {\bar{u}}_{i}^{\left(0\right)}}{\partial \eta }{|}_{\eta =0}=0,$$

(43) $${\bar{u}}_{i}^{\left(0\right)}{|}_{\eta =1}={\bar{u}}_{s}^{\left(0\right)}{|}_{Z=0},$$

(44) $$-\frac{\partial {\bar{u}}_{i}^{\left(0\right)}}{\partial \eta }{|}_{\eta =1}-\alpha {\left[-\frac{\partial {\bar{u}}_{s}^{\left(0\right)}}{\partial Z}\right]}^{n}{|}_{Z=0}=\bar{\kappa }\frac{I_{1}\left(\bar{\kappa }\right)}{I_{0}\left(\bar{\kappa }\right)}\frac{d{\bar{\phi }}_{i}^{\left(0\right)}}{d\chi },$$

(45) $${\bar{u}}_{s}^{\left(0\right)}{|}_{Z=1}=0,$$

(46) $$\theta_{i}^{\left(0\right)}=\theta_{s}^{\left(0\right)}=0\;\mathrm{at}\;\chi =0,1$$

and:

(47) $${\bar{\phi }}_{i}^{\left(0\right)}{|}_{\chi =0}=1\;\mathrm{and}\;{\bar{\phi }}_{i}^{\left(0\right)}{|}_{\chi =1}=0.$$

The solution for the electric potential of the conducting fluid is determined from Equations (41) and (47), and it is given by:

(48) $${\bar{\phi }}_{i}^{\left(0\right)}=1-\chi .$$

The velocity profiles for the inner and surrounding fluids are readily obtained from Equations (37), (38) and (42)–(45), respectively, as:

(49) $${\bar{u}}_{i}^{\left(0\right)}=\left[1-\frac{I_{0}\left(\bar{\kappa }\eta \right)}{I_{0}\left(\bar{\kappa }\right)}\right]+\frac{\delta^{N}}{\xi \left(1-N\right)}\left[{\left(1+\xi \right)}^{1-N}-1\right]$$

and:

(50) $${\bar{u}}_{s}^{\left(0\right)}=\frac{\delta^{N}}{\xi \left(1-N\right)}\left[{\left(1+\xi \right)}^{1-N}-{\left(1+\xi Z\right)}^{1-N}\right],$$

where $\delta =2\bar{\kappa }I_{1}\left(\bar{\kappa }\right)/\alpha I_{0}\left(\bar{\kappa }\right)$ and N=1/n.

Clearly, at the zeroth order, the dimensionless velocity profile of the inner conducting fluid is composed of two terms: the first term denotes the EOF, whereas the second term reflects the influence of the surrounding fluid, which acts as a lubricant. Note that in the limit N→1, the Newtonian fluid case is recovered. Under this last condition, the dimensionless velocity profile of the inner fluid is given by:

(51) $${\bar{u}}_{i}^{\left(0\right)}=\left\{1-\frac{I_{0}\left(\bar{\kappa }\eta \right)}{I_{0}\left(\bar{\kappa }\right)}+\frac{2\bar{\kappa }}{\mu_{r}}\frac{I_{1}\left(\bar{\kappa }\right)}{I_{0}\left(\bar{\kappa }\right)}\ln\left(\frac{R_{2}}{R_{1}}\right)\right\}$$

and the corresponding dimensionless velocity profile (for N→1) of the surrounding fluid is obtained as:

(52) $${\bar{u}}_{s}^{\left(0\right)}=\frac{2\bar{\kappa }}{\mu_{r}}\frac{I_{1}\left(\kappa \right)}{I_{0}\left(\kappa \right)}\ln\left(\frac{1+\xi }{1+\xi Z}\right)$$

The magnitude of the dimensionless velocity, at the zeroth order, evaluated at the interface between both fluids is:

(53) $${\bar{u}}_{i}^{\left(0\right)}={\bar{u}}_{s}^{\left(0\right)}=\frac{2\bar{\kappa }}{\mu_{r}}\frac{I_{1}\left(\bar{\kappa }\right)}{I_{0}\left(\bar{\kappa }\right)}\ln\left(R_{r}\right).$$

The solution for the dimensionless temperature profile in the capillary is obtained from Equations (39) and (40) and is given by (see Appendix A or the details):

(54) $$\theta_{i}^{\left(0\right)}\approx \theta_{s}^{\left(0\right)}=\frac{\bar{\alpha }\beta_{i}}{2k_{T}\left(1+\xi \right)}\left\{1-\frac{\exp\left(m_{2}\chi \right)\left[1-\exp\left(m_{1}\right)\right]-\exp\left(m_{1}\chi \right)\left[1-\exp\left(m_{2}\right)\right]}{\exp\left(m_{2}\right)-\exp\left(m_{1}\right)}\right\}.$$

The above equation defines the temperature distribution at the leading order for both fluids. Parameters $m_{1}$ and $m_{2}$ are defined in Appendix A.

An order-of-magnitude estimate of the incremental temperature rise can be determined from the first factor on the right-hand side of Equation (54). In this sense, the temperature rise can be estimated from $\theta_{i}^{\left(0\right)}\approx \theta_{s}^{\left(0\right)}=\bar{\alpha }\beta_{i}/2k_{T}\left(1+\xi \right)$, which in physical units can be expressed as:

(55) $$T_{i,s}\propto T_{0}+\frac{\sigma_{0}E_{0}^{2}R_{1}^{2}}{h_{eq}R_{2}}.$$

The above relationship clearly indicates how the temperature in the capillary can be modulated depending on the assumed values of the physical parameters.

### 3.2. The $O\left(\gamma_{\mu }\right)$ Solution

The $O\left(\gamma_{\mu }\right)$ problem is defined by the following set of equations:

(56) $$\frac{\partial {\bar{u}}_{i}^{\left(1\right)}}{\partial \chi }+\frac{1}{\eta }\frac{\partial }{\partial \eta }\left(\eta {\bar{v}}_{i}^{\left(1\right)}\right)=0,$$

(57) $$\frac{1}{\eta }\frac{\partial }{\partial \eta }\left[\eta \frac{\partial {\bar{u}}_{i}^{\left(1\right)}}{\partial \eta }-\eta \theta_{i}^{\left(0\right)}\frac{\partial {\bar{u}}_{i}^{\left(0\right)}}{\partial \eta }\right]-{\bar{\kappa }}^{2}\frac{I_{0}\left(\bar{\kappa }\eta \right)}{I_{0}\left(\bar{\kappa }\right)}\frac{d{\bar{\phi }}_{i}^{\left(1\right)}}{d\chi }-\frac{d{\bar{p}}_{i}^{\left(1\right)}}{d\chi }=0,$$

(58) $$\begin{array}{c} -\frac{\partial }{\partial Z}\left[n\frac{\partial {\bar{u}}_{s}^{\left(1\right)}}{\partial Z}\frac{{\left(-\frac{\partial u_{s}^{\left(0\right)}}{\partial Z}\right)}^{n}}{\frac{\partial {\bar{u}}_{s}^{\left(0\right)}}{\partial Z}}\left(1+\xi Z\right)-\Gamma_{a}\theta_{s}^{\left(0\right)}{\left(-\frac{\partial {\bar{u}}_{s}^{\left(0\right)}}{\partial Z}\right)}^{n}\left(1+\xi Z\right)\right]-\Lambda \frac{d{\bar{p}}_{s}^{\left(1\right)}}{d\chi }\left(1+\xi Z\right)=0 \end{array}$$

and:

(59) $$\frac{d}{d\chi }\left(\frac{d{\bar{\phi }}_{i}^{\left(1\right)}}{d\chi }+\Gamma_{\sigma }\theta_{i}^{\left(0\right)}\frac{d{\bar{\phi }}_{i}^{\left(0\right)}}{d\chi }\right)=0.$$

At this order, the velocity profiles for both fluids only depend on the zeroth-order temperature; accordingly, it is not necessary to consider the energy equation for terms of $O\left(\gamma_{\mu }\right)$. Furthermore, we can neglect small pressure differences due to the curvature effects associated with the surface tension. In this case, the pressure gradients are equal in both fluids [21]. Therefore, we assume that $d{\bar{p}}_{i}^{\left(1\right)}/d\chi =d{\bar{p}}_{s}^{\left(1\right)}/d\chi =d{\bar{p}}^{\left(1\right)}/d\chi$.

The boundary conditions for Equations (56)–(59) are as follows:

(60) $$\frac{\partial {\bar{u}}_{i}^{\left(1\right)}}{\partial \eta }{|}_{\eta =0}=0,$$

(61) $${\bar{v}}_{i}^{\left(1\right)}=0\;\mathrm{at}\;\eta =0,1,$$

(62) $${\bar{u}}_{i}^{\left(1\right)}{|}_{\eta =1}={\bar{u}}_{s}^{\left(1\right)}{|}_{Z=0},$$

(63) $${\bar{p}}^{\left(1\right)}{|}_{\chi =0}={\bar{p}}^{\left(1\right)}{|}_{\chi =1}=0,$$

(64) $$\begin{array}{c} {\left.{\left.\theta_{i}^{\left(0\right)}\frac{\partial {\bar{u}}_{i}^{\left(0\right)}}{\partial \eta }\right|}_{\eta =1}-\frac{\partial {\bar{u}}_{i}^{\left(1\right)}}{\partial \eta }\right|}_{\eta =1}+{\left.\alpha \Gamma_{a}\theta_{s}^{\left(0\right)}{\left(-\frac{\partial {\bar{u}}_{s}^{\left(0\right)}}{\partial Z}\right)}^{n}\right|}_{Z=0}-{\left.\alpha n\frac{\partial {\bar{u}}_{s}^{\left(1\right)}}{\partial Z}\frac{{\left(-\frac{\partial {\bar{u}}_{s}^{\left(0\right)}}{\partial Z}\right)}^{n}}{\frac{\partial {\bar{u}}_{s}^{\left(0\right)}}{\partial Z}}\right|}_{Z=0}=\left.\bar{\kappa }\frac{I_{1}\left(\bar{\kappa }\right)}{I_{0}\left(\bar{\kappa }\right)}\frac{d{\bar{\phi }}_{i}^{\left(1\right)}}{d\chi },\right. \end{array}$$

(65) $${\bar{u}}_{s}^{\left(1\right)}{|}_{Z=1}=0$$

and:

(66) $${\bar{\phi }}_{i}^{\left(1\right)}{|}_{\chi =0}={\bar{\phi }}_{i}^{\left(1\right)}{|}_{\chi =1}=0.$$

Notably, the boundary condition for the stresses at the interface is influenced by the temperature effects, which is reflected by $\theta_{i}^{\left(0\right)}$ and $\theta_{s}^{\left(0\right)}$, included in the first and third terms on the left-hand side of Equation (64), respectively. Additionally, the term on the right-hand side of this equation, $d{\bar{\phi }}_{i}^{\left(1\right)}/d\chi$, depends on the temperature field, as shown in Equation (59).

By applying standard methods, the solution for the above system of equations for the electric potential and velocity fields is the following:

$$\begin{array}{c} {\bar{\phi }}_{i}^{\left(1\right)}=\frac{\bar{\alpha }\beta_{i}}{2k_{T}\left(1+\xi \right)}\frac{\Gamma_{\sigma }F_{0}}{\exp\left(m_{2}\right)-\exp\left(m_{1}\right)}\left\{\frac{\left[1-\exp\left(m_{1}\right)\right]}{m_{2}}\left[\chi \exp\left(m_{2}\right)-\exp\left(m_{2}\chi \right)\right]\right.\\ \left.+\frac{\left[1-\exp\left(m_{2}\right)\right]}{m_{1}}\left[\exp\left(m_{1}\chi \right)-\chi \exp\left(m_{1}\right)\right]+\frac{\exp\left(m_{2}\right)-\exp\left(m_{1}\right)}{m_{2}}\left(1-\chi \right)\right\} \end{array}$$

(67) $$\begin{array}{cc} {\bar{u}}_{i}^{\left(1\right)}= & \frac{d{\bar{p}}^{\left(1\right)}}{d\chi }\left\{\frac{1}{4}\left(\eta^{2}-1\right)+\frac{\delta^{N-1}}{2\alpha \xi \left(n-1\right)}\left[\frac{1}{{\left(1+\xi \right)}^{N-1}}-1\right]\right.\\ & \left.-\frac{N\Lambda \delta^{N-1}}{\left(6n^{2}-8n+2\right)\xi^{2}}\left[\frac{\left(n-1\right)\xi^{2}+2\left(n-1\right)\xi -2n}{{\left(1+\xi \right)}^{N-1}}+2n\right]\right\}\\ & -\frac{d{\bar{\phi }}_{i}^{\left(1\right)}}{d\chi }\left\{\frac{2\bar{\kappa }\delta^{N-1}}{\alpha \xi \left(n-1\right)}\frac{I_{1}\left(\bar{\kappa }\right)}{I_{0}\left(\bar{\kappa }\right)}\left[\frac{1}{{\left(1+\xi \right)}^{N-1}}-1\right]-\frac{I_{0}\left(\bar{\kappa }\eta \right)}{I_{0}\left(\bar{\kappa }\right)}+1\right\}\\ & +\theta^{\left(0\right)}\left\{{\bar{u}}_{i}^{\left(0\right)}+\frac{\delta^{N}}{\xi \left(1-N\right)}\left[{\left(\xi +1\right)}^{1-N}\left(\frac{\Gamma_{a}}{n}-1\right)-\frac{\Gamma_{a}}{n}+1\right]\right\}. \end{array}$$

(68) $$\begin{array}{cc} {\bar{u}}_{s}^{\left(1\right)} & =\frac{d{\bar{p}}^{\left(1\right)}}{d\chi }\left\{\frac{N\Lambda {\left(\frac{\delta }{1+\xi Z}\right)}^{N-1}}{\left(6n^{2}-8n+2\right)\xi^{2}}\left[Z\xi \left(n-1\right)\left(Z\xi +2\right)-\left(n-1\right)\xi \left(\xi +2\right)\right]\right.\\ & \left.+\frac{\delta^{N-1}}{2\alpha \xi \left(n-1\right)}\left[\frac{1}{{\left(1+\xi \right)}^{N-1}}-\frac{1}{{\left(1+\xi Z\right)}^{N-1}}\right]\right\}\\ & -\frac{d{\bar{\phi }}_{i}^{\left(1\right)}}{d\chi }\left\{\frac{1}{{\left(1+\xi \right)}^{N-1}}-\frac{1}{{\left(1+\xi Z\right)}^{N-1}}\right\}\frac{2\bar{\kappa }\delta^{N-1}}{\alpha \xi \left(n-1\right)}\frac{I_{1}\left(\bar{\kappa }\right)}{I_{0}\left(\bar{\kappa }\right)}\\ & +\frac{N\Gamma_{a}\delta^{N}}{\xi \left(1-N\right)}\theta_{0}\left[{\left(1+\xi \right)}^{1-N}-{\left(1+\xi Z\right)}^{1-N}\right], \end{array}$$

where $F_{0}=\bar{\alpha }\beta_{i}/2k_{T}\left(1+\xi \right)$. In Equations (67) and (68), the pressure gradient $d{\bar{p}}^{\left(1\right)}/d\chi$ is unknown. Therefore, to determine the pressure gradient, we substitute ${\bar{u}}_{i}^{\left(1\right)}$ into the continuity Equation (56), and after integrating the previous result in the radial direction and by applying the impermeability boundary conditions, Equation (61), we obtain the solution for ${\bar{p}}_{1}$:

(69) $$\begin{array}{c} {\bar{p}}^{\left(1\right)}=-\frac{C_{2}}{C_{1}}F_{0}\left\{\chi -\frac{1}{\exp\left(m_{2}\right)-\exp\left(m_{1}\right)}\left(\frac{\exp\left(m_{2}\chi \right)}{m_{2}}\left[1-\exp\left(m_{1}\right)\right]\right.\right.\\ \left.\left.-\frac{\exp\left(m_{1}\chi \right)}{m_{1}}\left[1-\exp\left(m_{2}\right)\right]\right)\right\}+C_{3}\chi +C_{4} \end{array}$$

and the corresponding pressure gradient is obtained as:

(70) $$\frac{d{\bar{p}}^{\left(1\right)}}{d\chi }=-\frac{C_{2}}{C_{1}}\theta_{0}+C_{3},$$

where $C_{1}$–$C_{4}$ are known parameters, which are given in Appendix B.

Once the velocity and pressure profiles are known, the volumetric flow rate can be obtained.

### 3.3. Volumetric Flow Rate

The dimensionless volumetric flow rates for both the inner (${\bar{Q}}_{i}=Q_{i}/Q_{c}$) and the outer (${\bar{Q}}_{s}=/Q_{c}$) fluids can now be determined; here, $Q_{c}=\pi u_{c}R_{1}^{2}$ is the characteristic volumetric flow rate. Therefore, we have that:

(71) $$\begin{array}{c} {\bar{Q}}_{i}=\int_{0}^{1}2{\bar{u}}_{i}\eta d\eta =\langle {\bar{u}}_{i}^{\left(0\right)}\rangle +2\gamma_{\mu }\left\{\frac{1}{2}\frac{d{\bar{p}}^{\left(1\right)}}{d\chi }k_{1}+\theta^{\left(0\right)}k_{2}-\frac{d{\bar{\phi }}_{i}^{\left(1\right)}}{d\chi }\frac{k_{3}}{\bar{\kappa }}\right\} \end{array}$$

and:

(72) $$\begin{array}{c} {\bar{Q}}_{s}=2\xi \int_{0}^{1}{\bar{u}}_{s}\left(1+\xi Z\right)dZ=\left(1+R_{r}\right)\xi \langle {\bar{u}}_{s}^{\left(0\right)}\rangle +2\gamma_{\mu }\xi \left\{\theta^{\left(0\right)}k_{5}-\frac{d{\bar{p}}^{\left(1\right)}}{d\chi }k_{6}-\frac{d{\bar{\phi }}_{i}^{\left(1\right)}}{d\chi }k_{7}\right\}. \end{array}$$

In Equations (71) and (72), the second terms on the right-hand sides in both equations represent the influence of thermal effects on the volumetric flow rate. In the same equations, the expressions for $\langle {\bar{u}}_{i}^{\left(0\right)}\rangle$ and $\langle {\bar{u}}_{s}^{\left(0\right)}\rangle$ are defined in Appendix A. Parameters $k_{1}$–$k_{10}$ are presented in Appendix C.

## 4. Results and Discussion

For estimating the values of the dimensionless parameters used in this work, we consider values of physical and geometric parameters that are typical in EOFs, as shown in Table 1. Consequently, the following values for the dimensionless parameters were used in the calculations: $\bar{\kappa }=40$, $\gamma_{\mu }=0.01,$ $\Gamma_{a}=4.91,$ $\Gamma_{\sigma }=0.49,$ $k_{r}=1.1,$ $\mu_{r}=10,$ $R_{r}=2,$ Pe=0.05 and $k_{T}=0.0023$.

**Figure 2:**
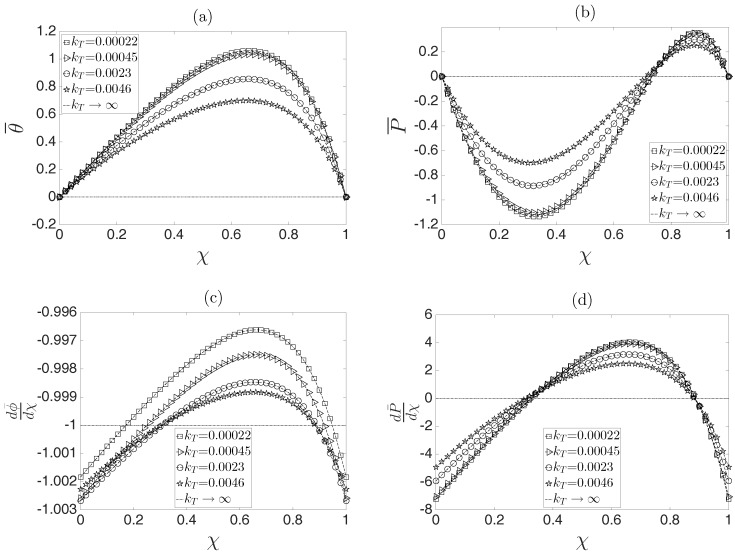
Influence of the dimensionless parameter $K_{T}$ on the dimensionless (**a**) temperature, (**b**) pressure, (**c**) electric field and (**d**) pressure gradient.

In Figure 2a–d, dimensionless profiles for temperature, pressure, electric potential gradient and pressure gradient as functions of the dimensionless axial coordinate χ and different values of $k_{T}$ are shown. Figure 2a clearly shows that for increasing values of this parameter, the heat dissipation through the microcapillary system is larger; hence, lower values of temperature are obtained. If high values of $h_{eq}$ are considered, which means that a very high cooling is present on the surface of the capillary such that $k_{T}\to \infty$, it is possible to obtain the isothermal case, as represented by the dashed line, i.e., $\bar{\theta }=0$. This behavior can be explained because the first term on the right-hand side of Equation (54) determines the magnitude of the dimensionless temperature according to $\theta_{0}∼\bar{\alpha }\beta_{i}/2k_{T}\left(1+\xi \right)$. From a physical perspective, the magnitude of the fluid temperature is modulated according to $T_{i}=T_{s}∼T_{0}+\sigma_{0}E_{0}^{2}R_{1}/h_{eq}R_{2}$. Evidently, depending on the values of the parameters involved in the aforementioned relationships, the magnitude of the fluid temperature will change.

Due to the dependencies of the viscosities of both fluids and the thermal conductivity of the inner fluid on temperature, an induced pressure is generated along the microcapillary, as shown in Figure 2b. That is to guarantee the mass conservation of the flow in the $O(\gamma_{\mu })$ problem defined by Equations (56)–(59), where temperature effects are taken into account. In the case of Figure 2b, when $k_{T}=0.00022$ (a lower heat flux at the microchannel wall), the temperature effects are maximum, generating more representative pressure values. Conversely, for $k_{T}\to \infty$ (a higher heat flux at the microchannel wall), the pressure distribution disappears, which is consistent with the comment made in the previous paragraph. Meanwhile, the electric field and pressure gradient distributions that are necessary to solve the correction of the velocity profiles given in Equations (67)–(68) by temperature effects are shown in Figure 2c,d. It is clear that in the case of $k_{T}\to \infty$, Joule heating effects are minimized on the flow along the microcapillary, indicating that the pressure distribution is constant and equal to zero. For the same conditions described previously, the electric field is constant, i.e., $d\bar{\phi }/d\chi =-1$, recovering the case of an EOF where the physical properties are assumed to be constant. In contrast, it can be appreciated that the variation of physical properties modifies the magnitude of the electric field along the microcapillary in a slight manner. Here, note that the pressure gradient has the same dependence on χ as the temperature field, which is in accordance with Equation (70), i.e., $d\bar{P}/d\chi ∼\bar{\theta }$.

**Figure 3:**
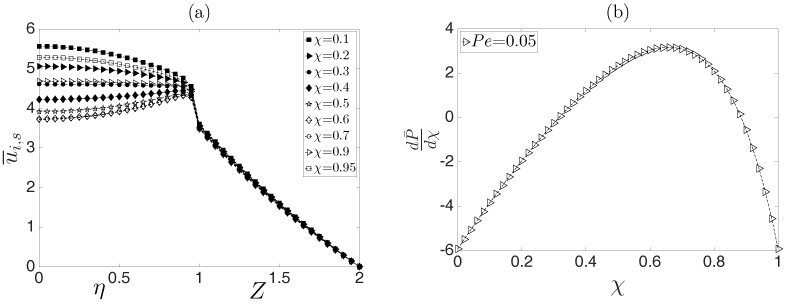
(**a**) Dimensionless velocity profiles, evaluated at different values of the coordinate χ, and (**b**) the corresponding pressure gradient along the microcapillary.

A consequence of the Joule heating effect is shown in Figure 3. It is evident that when the physical properties are temperature dependent, the flow is no longer developed. In such a case, when the dimensionless pressure gradient is positive, dP/dχ>0, the velocity profiles are convex, whereas for negative values, dP/dχ<0, they are concave. For dP/dχ=0, the plug-like electroosmotic velocity profile is recovered. In fact, the dimensionless fluid velocity for the inner and surrounding fluids is affected by the dimensionless pressure and electric gradients, together with the temperature field, as shown in Equations (67) and (68). If the above variables are absent, then there are no corrections to the velocity profiles by the temperature effects. In the same context, as shown in the same figure, the velocity profiles of the surrounding fluid are only affected in a very weak manner by temperature effects; however, we anticipate that this variable is more notably affected due to the viscosities of both fluids, as will be shown in the following paragraphs.

**Figure 4:**
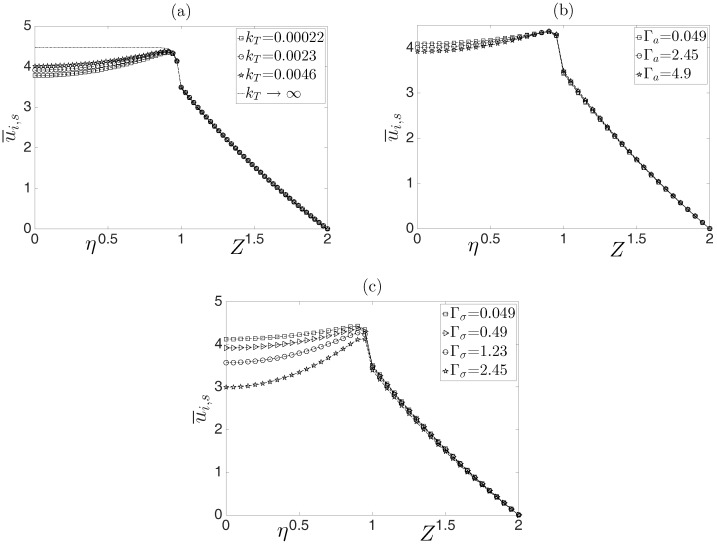
Dimensionless velocity profiles for the inner and surrounding fluids as a function of the dimensionless radial coordinates η and *Z*. (**a**) Effect of the parameter $k_{T}$; (**b**) effect of the parameter $\Gamma_{a}$; (**c**) effect of the parameter $\Gamma_{\sigma }$.

Figure 4 shows the dimensionless velocity profiles of the flow, ${\bar{u}}_{i}$ and ${\bar{u}}_{s}$, evaluated at an arbitrary dimensionless coordinate χ=0.5 as a function of the dimensionless transverse coordinates η for the inner fluid and *Z* for the surrounding fluid. It is evident that the influence of temperature only affects the inner fluid. The effect of the parameter $k_{T}$ is shown in Figure 4a. Increasing values of this parameter indicate that the convected heat through the external surface of the capillary is larger, which minimizes the Joule heating. Therefore, the variable effects in the physical properties due to temperature changes are very weak, which is confirmed by the case of $k_{T}\to \infty$, yielding a uniform temperature through the microcapillary. Therefore, no variations in the physical properties exist. Note that the velocity profiles of the surrounding fluid are not affected by temperature changes obtained by the different values of $k_{T}$. Figure 4b shows the influence of the dimensionless parameter $\Gamma_{a}$, which relates the parameters *a* and $B_{\mu }$ and measures the sensitivity of the consistency index of the non-Newtonian fluid and of the viscosity of the Newtonian fluid to temperature variations. In the same context, in Figure 4c, the effect of the parameter $\Gamma_{\sigma }$ on the velocity profiles is plotted. In this case, this parameter is the ratio of the sensitivity of the electrical conductivity to temperature variations. As observed, for decreasing values of $\Gamma_{\sigma }$, the velocity gradient through the transversal section of the inner fluid is weaker compared with increasing values of this parameter. The above allows us to identify that the electrical conductivity of the inner fluid has a stronger effect on the flow field compared with that caused by its viscosity.

**Figure 5:**
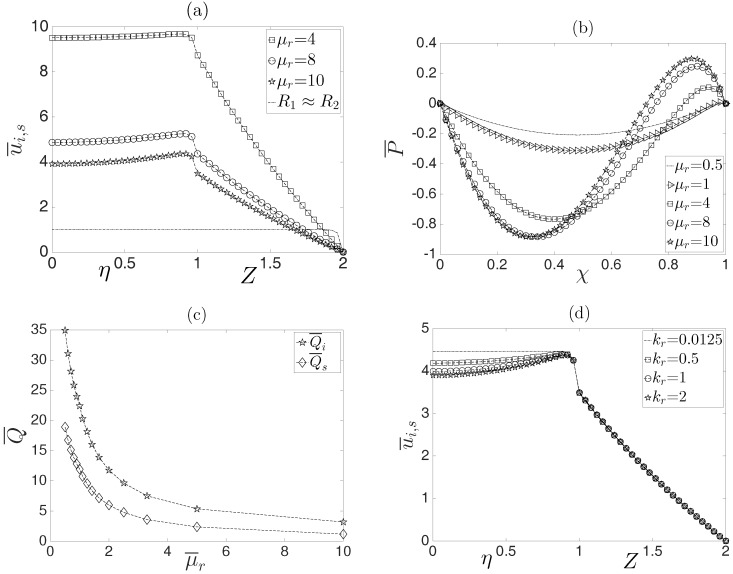
Influence of the viscosity ratio on the dimensionless velocity profiles (**a**) and dimensionless pressure (**b**). Volumetric flow rate as a function of the viscosity ratio (**c**) and the effect of the thermal conductivity ratio between the surrounding and inner fluids on the dimensionless velocity profiles (**d**).

The effect of the viscosity ratio on the velocity profile is shown in Figure 5a for values of $\mu_{r}=4,8,10$. Because of the complexity of the obtained velocity solutions, a simplified analysis can be performed in the limit of n→1. As predicted by Equation (53), for fixed $\bar{\kappa },R_{1}$ and $R_{2}$, the velocity at the interface between both fluids, at the leading order, varies according to ${\bar{u}}_{i,s}∼\mu_{r}^{-1}$; it is evident that for increasing values of $\mu_{r}$, the average velocity of the two fluids diminishes. In addition, in Figure 5a, we have plotted the case when the inner fluid fills the capillary and is isothermal, i.e., $R_{1}\approx R_{2}$ (dashed-dotted line). In such a case, the classical Helmholtz–Smoluchowski velocity is recovered [3]; the above can be inferred from Equation (51), yielding ${\bar{u}}_{i}^{\left(0\right)}=1-I_{0}\left(\bar{\kappa }\eta \right)/I_{0}\left(\bar{\kappa }\right)$. Besides, in this case, the velocity of the surrounding fluid is zero (see Equation (52)), ${\bar{u}}_{s}^{\left(0\right)}=0$. Moreover, examination of Figure 5b shows that the pressure profiles strongly depend on $\mu_{r}$. As shown in this figure, greater values of $\mu_{r}$ yield greater variations in the pressure field along the capillary, which in turn will modify the flow field. For relatively low values, i.e., $\mu_{r}<1$, the induced pressure tends to disappear, yielding a plug-like velocity profile in the inner fluid. Figure 5d shows the effect of the thermal conductivity ratio of the inner fluid to the surrounding fluid; as shown, when $k_{r}\to 0$, a plug-like velocity profiles is obtained. This result indicates that the thermal conductivity of the surrounding fluid is greater than the thermal conductivity of the inner fluid, which causes the heat generated by Joule heating in the inner fluid to dissipate more quickly to the microcapillary wall, thereby causing the physical properties of the fluid to change slightly with temperature.

The influence of the viscosity ratio $\mu_{r}$ on the volumetric flow rate of the inner and surrounding fluids is shown in Figure 5c. As shown, when the consistency index of the non-Newtonian fluid is greater than the viscosity of the Newtonian fluid, the volumetric flow rate decreases for both fluids. This result occurs because the conducting Newtonian fluid has to drag a more viscous fluid.

**Figure 6:**
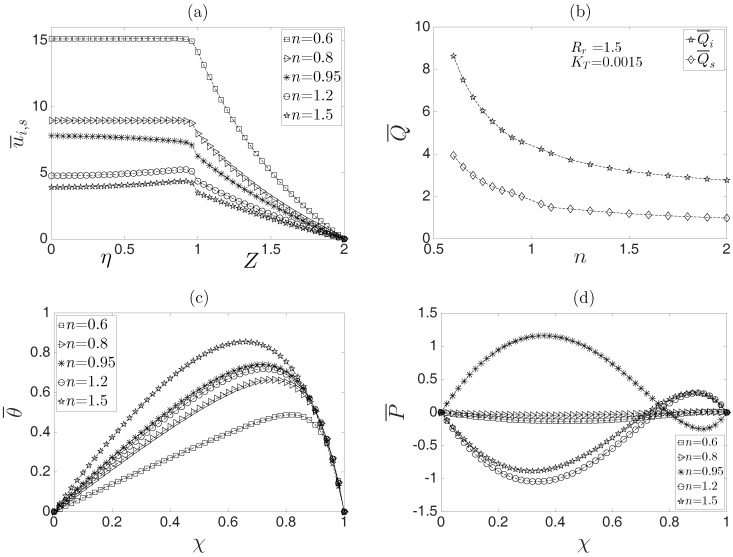
Behavior of the electroosmotic flow with respect to the power-law index *n*: (**a**) dimensionless velocity profiles, (**b**) dimensionless flow rate, (**c**) dimensionless temperature and (**d**) dimensionless pressure.

Figure 6a shows the dimensionless velocity profiles as a function of the dimensionless radial coordinate for different values of the power-law index at an arbitrary axial position χ=0.5. It is clear that the inner conducting fluid drags the surrounding fluid, which has a non-Newtonian behavior. In this sense, the coupling of two immiscible fluids in the EOF yields higher values of the dimensionless velocity for pseudoplastic surrounding fluids than dilatant surrounding fluids due to the shear thinning effects at the interface between both fluids for values of n<1, where the flow rate is significantly higher (Figure 6b).

Thus, for fluids with n<1 (pseudoplastic effect of the surrounding fluid), the Joule heating effect diminishes, as shown in Figure 6c, causing a decrease in the temperature in the microcapillary. In addition, the influence of the power-law index on the induced pressure field is shown in Figure 6d; depending on the assumed values of *n*, the pressure gradient significantly varies. Of course, the behavior shown in this figure will cause convex or concave velocity profiles in the inner fluid.

**Figure 7:**
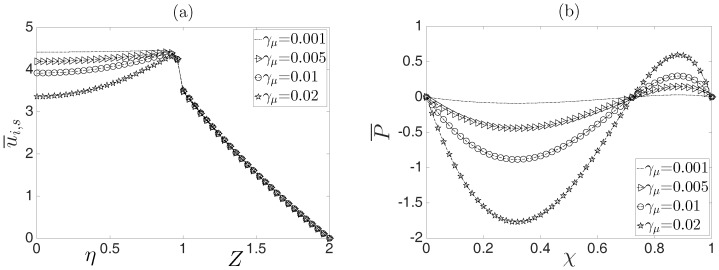
Behavior of the electroosmotic flow: (**a**) dimensionless velocity profile, evaluated at χ=0.5, and (**b**) dimensionless pressure along the microcapillary.

In Figure 7a, velocity profiles as a function of the dimensionless transverse coordinates η and *Z* are plotted, and the axial position is χ=0.5 for different values of the parameter $\gamma_{\mu }$, which reflects the influence of variations of physical properties by temperature gradients. For $\gamma_{\mu }\to 0$, the plug-like velocity profile is recovered. However, the Joule heating effect is more representative in the EOF when $\gamma_{\mu }$ increases. In Figure 4b, for the same values of the parameter $\gamma_{\mu }$, the induced pressure distribution along the χ−coordinate is shown. It is clear that any variation of $\gamma_{\mu }$ yields a change in the flow field. As shown, at χ=0.5, for increasing values of $\gamma_{\mu }$, larger pressure gradients are obtained, which correspond to the velocity profiles in Figure 7a, where larger gradients in the velocity profiles are observed.

One aspect that should be observed is that the velocity profiles for the inner fluid (see Figure 3, Figure 4, Figure 5, Figure 6, Figure 7) near the interface between both fluids resemble the typical Helmholtz–Smoluchowski velocity that is present in a purely electroosmotic flow in microchannels. As can be seen, strong gradients appear in the region where the electric double layer exists. The above can be explained by considering the momentum equation for the inner fluid, Equation (19). In the case of $\bar{\kappa }\to \infty$, the viscous term (second order derivative) would be multiplied by $1/\bar{\kappa }$ and thus corresponds to a “singular perturbation” [18], causing strong gradients of velocity.

**Figure 8:**
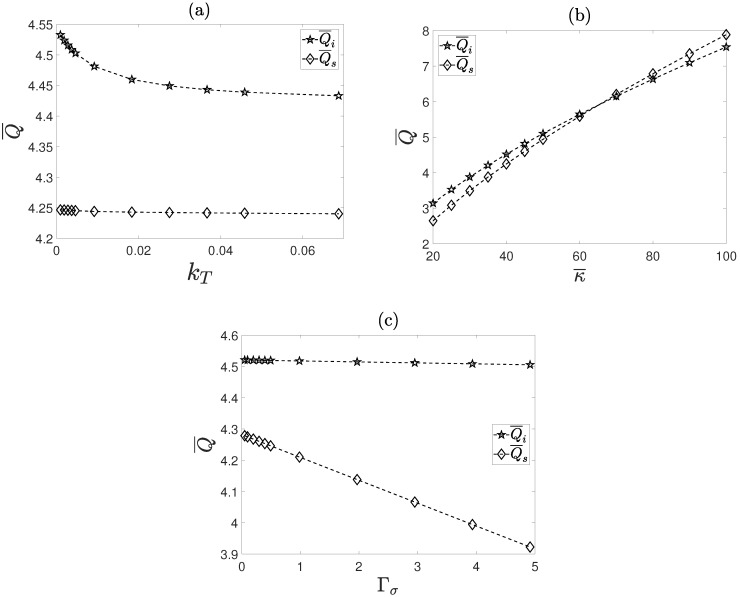
Volumetric flow rates for the inner and surrounding fluids: (**a**) effect of the parameter $k_{T}$; (**b**) effect of the parameter $\bar{\kappa }$; (**c**) effect of the parameter $\Gamma_{\sigma }$.

The volumetric flow rate $\bar{Q}$ as a function of the dimensionless parameters $k_{T}$, $\bar{\kappa }$ and $\Gamma_{\sigma }$ is shown in Figure 8a–c. It can be seen that the effect of the heat dissipated through the external surface of the microcapillary, reflected in the parameter $k_{T}$, affects to a greater extent the volumetric flow rate of the inner fluid, ${\bar{Q}}_{i}$, in comparison with the flow rate of the surrounding fluid, ${\bar{Q}}_{s}$. In the same figure, for $k_{T}\to 0$, the volumetric flow rate ${\bar{Q}}_{i}$ is increased. An explanation of the above could be as follows: the fluid temperature varies according to $\theta_{0}∼k_{T}^{-1}$, which in turn diminishes the viscosity of the fluids, causing larger volumetric flow rates. In Figure 8b, the volumetric flow rates of the inner and of the surrounding fluid are plotted as functions of the electrokinetic parameter $\bar{\kappa }$. It should be noted that $\bar{Q}$ is a monotonically increasing function in terms of the electrokinetic parameter. It should be noted that for $\bar{\kappa }>60$, the volumetric flow rate ${\bar{Q}}_{s}$ becomes greater than ${\bar{Q}}_{i}$, indicating that the inner fluid has a great ability of dragging the surrounding fluid. Finally, the effect of the electric sensitivity to temperature variations on the volumetric flow rate is shown in Figure 8c. This sensitivity is represented in the parameter $\Gamma_{\sigma }$. As can be appreciated, for increasing values of $\Gamma_{\sigma }$, the inner fluid flow decreases in a light manner, however originating a strong decrement in the surrounding fluid flow. From a physical point of view, increasing values of $\Gamma_{\sigma }$ mean that the inner fluid behaves as a weakly electrical conductor and, accordingly, diminishing the electroosmotic force.

## 5. Conclusions

In this work, we delineate the effects of considering temperature-dependent physical properties due to Joule heating in an EOF in a microcapillary with two immiscible fluids. We have considered an inner column of a conducting Newtonian liquid surrounded by an annular non-conducting liquid with non-Newtonian behavior, whose rheological model follows a power-law. In this regard, we have shown that taking the Joule heating effect into account strongly affects the hydrodynamic and electric fields; thus, considering this effect is very important for predicting characteristics in the non-isothermal EO of immiscible fluids. In particular, the viscosity coefficients of both fluids and the electrical conductivity of the conducting fluid were assumed to be temperature dependent. From the principal obtained results, we showed that the volumetric flow through the microcapillary depends on several dimensionless parameters: the competition between the consistency index, $\mu_{r}$, of the non-Newtonian fluid to the viscosity of the Newtonian fluid, the power-law index *n*, the equivalent Biot number $k_{T}$ and the thermal conductivity ratio $k_{r}$ of both fluids, with $\mu_{r}$, *n* and $\bar{\kappa }$ being the most significant parameters that greatly affect the volumetric flow rate. Moreover, when a high dissipation of heat through the external surface of the microcapillary is assumed, the isothermal case is recovered.

Future work concerns the improvement of the present analysis by considering that the interface between both fluids is nonuniform; in addition, another possibility to be studied is the analysis of the associated hydrodynamic stability caused by thermal effects.

## Figures and Tables

**Table 1:** Physical and geometrical parameters used for estimating the dimensionless parameters used in the present analysis.

Parameter	Value	Units	Definition
*a*	<$10^{-1}$	$K^{-1}$	sensitivity constant for the consistency index
$B_{\mu }$	1713	K	sensitivity constant for the viscosity
$B_{\sigma }$	∼$10^{-2}$	$K^{-1}$	sensitivity constant for the electrical conductivity
$E_{0}$	∼$10^{4}$	V·$m^{-1}$	external electric field
$h_{eq}$	100	W·$m^{-2}$·$K^{-1}$	equivalent heat transfer coefficient
$k_{i}$	0.609	W·$m^{-1}$·$K^{-1}$	thermal conductivity of the inner fluid
$k_{s}$	0.145	W·$m^{-1}$·$K^{-1}$	thermal conductivity of the surrounding fluid
$k_{w}$	1.5	W·$m^{-1}$·$K^{-1}$	thermal conductivity of the microcapillary wall
*L*	∼$10^{-3}$–$10^{-2}$	m	microcapillary length
$m_{0}$	∼$10^{-3}$	N·$m^{-2}$·$s^{n}$	consistency index evaluated at the reference temperature $T_{0}$
*n*	0.5–2		power-law index
$R_{1}$	50	μm	radius of the inner fluid
$R_{2}$	50	μm	external radius of the surrounding fluid
$T_{0}$	298	K	reference temperature
$\alpha_{i}$	3.96 $\times 10^{-6}$	$m^{2}$ · $s^{-1}$	thermal diffusivity of the inner fluid
$\alpha_{s}$	8.72 $\times 10^{-4}$	$m^{2}$ · $s^{-1}$	thermal diffusivity of the surrounding fluid
ζ	<−25	mV	zeta potential
$\mu_{0}$	∼$10^{-3}$	N·$m^{-2}$·s	viscosity evaluated at the reference temperature $T_{0}$
ϵ	7.08 $\times 10^{-10}$	C·$m^{-1}$ · $V^{-1}$	dielectric permittivity
$\sigma_{0}$	∼$10^{-2}$–$10^{-1}$	S·$m^{-1}$	electrical conductivity evaluated at the reference temperature $T_{0}$

## Appendix A

To determine the temperature fields for both fluids and considering that the temperature variations in the radial direction are very small, we can assume that, to a first approximation, $\theta_{i,0}\approx \theta_{s,0}\equiv \theta_{0}$; the above allows averaging the energy equations, Equations (39) and (40), in the radial direction. Therefore, we obtain the following equations:

(A1) $$\frac{d^{2}\theta_{0}}{d\chi^{2}}-S_{1}\frac{d\theta_{0}}{d\chi }+S_{2}{\left.\frac{\partial \theta_{i}}{\partial \eta }\right|}_{\eta =1}=-S_{3}$$

and:

(A2) $$\frac{d^{2}\theta_{0}}{d\chi^{2}}-A_{1}\frac{d\theta_{0}}{d\chi }-A_{2}\theta_{0}-A_{3}{\left.\frac{\partial \theta_{i}}{\partial \eta }\right|}_{\eta =1}=0,$$

where $S_{1}=Pe_{i}\langle {\bar{u}}_{i,0}\rangle /\beta_{i}$, $S_{2}=2/\beta_{i}^{2}$, $S_{3}=1/\beta_{i}$, $A_{1}=Pe_{s}\langle {\bar{u}}_{s,0}\rangle /\beta_{s}$, $A_{2}=2k_{T}\left(\xi +1\right)/\beta_{s}^{2}\left(1+R_{r}\right)$ and $A_{3}=2k_{r}\xi /\beta_{s}^{2}\left(1+R_{r}\right)$. Equations (A1) and (A2) must be solved simultaneously for θ and the temperature gradient ${\left.\partial \theta_{i}/\partial \eta \right|}_{\eta =1}$. Defining the operator D=d/dχ, the above equations can be rewritten as:

(A3) $$\left(D^{2}-S_{1}D\right)\theta_{0}+S_{2}{\left.\frac{\partial \theta_{i}}{\partial \eta }\right|}_{\eta =1}=-S_{3}$$

and:

(A4) $$\left(D^{2}-A_{1}D-A_{2}\right)\theta_{0}-A_{3}{\left.\frac{\partial \theta_{i}}{\partial \eta }\right|}_{\eta =1}=0.$$

Eliminating ${\left.\partial \theta_{i}/\partial \eta \right|}_{\eta =1}$ from Equations (A3) and (A4) yields:

(A5) $$\left[\left(A_{3}+S_{2}\right)D^{2}-\left(A_{3}S_{1}+A_{1}S_{2}\right)D-A_{2}S_{2}\right]\theta_{0}=-A_{3}S_{3},$$

which is a non-homogeneous differential equation. By solving Equation (A5) and applying the boundary conditions $\theta_{0}\left(\chi =0,1\right)=0$, it can easily be shown that the temperature distribution in the microcapillary becomes:

(A6) $$\theta_{0}=\frac{\bar{\alpha }\beta_{i}}{2k_{T}\left(1+\xi \right)}\left\{1-\frac{\exp\left(m_{2}\chi \right)\left[1-\exp\left(m_{1}\right)\right]-\exp\left(m_{1}\chi \right)\left[1-\exp\left(m_{2}\right)\right]}{\exp\left(m_{2}\right)-\exp\left(m_{1}\right)}\right\},$$

where:

$$m_{1}=\frac{F_{2}+{\left(F_{2}^{2}+4F_{1}F_{3}\right)}^{1/2}}{2F_{1}}\;\mathrm{and}\;m_{2}=\frac{F_{2}-{\left(F_{2}^{2}+4F_{1}F_{3}\right)}^{1/2}}{2F_{1}},$$

and:

$$F_{1}=A_{3}+S_{2},\;F_{2}=A_{3}S_{1}+A_{1}S_{2},\;F_{3}=A_{2}S_{2}\;\mathrm{and}\;F_{4}=A_{3}S_{3}.$$

Note that λ can easily be determined by substituting $\theta_{0}$ into Equation (A3) or Equation (A4). For simplicity, it is not necessary to show its definition. In addition, the average velocities $\langle {\bar{u}}_{i,0}\rangle$ and $\langle {\bar{u}}_{s,0}\rangle$ are determined using Equations (34)–(36), (49) and (50), obtaining:

(A7) $$\langle {\bar{u}}_{i,0}\rangle =\left[1-\frac{2}{\bar{\kappa }}\frac{I_{1}\left(\bar{\kappa }\right)}{I_{0}\left(\bar{\kappa }\right)}\right]+\frac{\delta^{N}}{\xi \left(1-N\right)}\left[{\left(1+\xi \right)}^{1-N}-1\right]$$

and:

(A8) $$\langle {\bar{u}}_{s,0}\rangle =\left(\frac{2}{1+R_{r}}\right)\left(\frac{\delta^{N}}{1-N}\right)\left[{\left(1+\xi \right)}^{1-N}\left(\frac{2+\xi }{2\xi }\right)-\frac{1-{\left(\xi \right)}^{-N}{\left(1+\xi \right)}^{2}{\left(\frac{1+\xi }{\xi }\right)}^{-N}}{\xi^{2}\left(N-2\right)}-k_{4}\right].$$

The above expressions for $\langle {\bar{u}}_{i,0}\rangle$ and $\langle {\bar{u}}_{s,0}\rangle$ represent the volumetric flow rate at zeroth order, i.e., when the physical properties are assumed to be constant.

## Appendix B

The expressions for $C_{1}$–$C_{4}$ presented in Section 3.2 are the following:

$$\begin{array}{c} C_{1}=\frac{\delta^{N-1}}{2\alpha \xi \left(n-1\right)}\left[1-\frac{1}{{\left(1+\xi \right)}^{N-1}}\right]\\ -N\Lambda \left[\frac{2n\delta^{N-1}+\left[\left(n-1\right)\xi^{2}+2\left(n-1\right)\xi -2n\right]{\left(\frac{\delta }{1+\xi }\right)}^{N-1}}{\left(6n^{2}-8n+2\right)\xi^{2}}\right], \end{array}$$

$$\begin{array}{c} C_{2}=\frac{\delta^{N}}{\xi \left(1-N\right)}\left\{2{\left(1+\xi \right)}^{1-N}-\frac{\Gamma_{\alpha }}{n}-2\right\}\\ +\frac{2{\bar{\kappa }}_{1}\delta^{N-1}}{\alpha \xi \left(n-1\right)}\frac{I_{1}\left({\bar{\kappa }}_{1}\right)}{I_{0}\left({\bar{\kappa }}_{1}\right)}\Gamma_{\mu }\left[1+\frac{1}{{\left(1+\xi \right)}^{N-1}}\right]+\Gamma_{\alpha }\frac{{\left(\xi +1\right)}^{1-N}\delta^{N}}{\xi \left(n-1\right)}, \end{array}$$

$$\begin{array}{c} C_{3}=\frac{C_{2}}{C_{1}}F_{0}\left\{1+\frac{1}{\exp\left(m_{2}\right)-\exp\left(m_{1}\right)}\left\{\frac{\left[1-\exp\left(m_{1}\right)\right]\left[1-\exp\left(m_{2}\right)\right]}{m_{2}}\right.\right.\\ \left.\left.+\frac{\left[1-\exp\left(m_{2}\right)\right]\left[\exp\left(m_{1}\right)-1\right]}{m_{1}}\right\}\right\}, \end{array}$$

$$\begin{array}{c} C_{4}=\frac{C_{2}}{C_{1}}\frac{F_{0}}{\exp\left(m_{2}\right)-\exp\left(m_{1}\right)}\left[\frac{1-\exp\left(m_{2}\right)}{m_{1}}-\frac{1-\exp\left(m_{1}\right)}{m_{2}}\right]. \end{array}$$

## Appendix C

The expressions for $k_{1}$–$k_{10}$ shown in Section 3.3 are given by the following relationships:

$$\begin{array}{c} k_{1}=\frac{\delta^{N-1}}{2\alpha \xi \left(n-1\right)}\left[\frac{1}{{\left(1+\xi \right)}^{N-1}}-1\right]\\ -\frac{\Lambda \delta^{N-1}}{\left(6n^{2}-8n+2\right)\xi^{2}}\left[2+\frac{\left[\left(n-1\right)\xi^{2}+2\left(n-1\right)\xi -2n\right]}{n{\left(1+\xi \right)}^{N-1}}\right], \end{array}$$

$$\begin{array}{c} k_{2}=\left\{\frac{1}{2}\frac{\delta^{N}\Gamma_{\alpha }N}{\xi \left(1-N\right)}\left[{\left(1+\xi \right)}^{1-N}-1\right]-\left[\frac{1}{\bar{\kappa }}\frac{I_{1}\left(\bar{\kappa }\right)}{I_{0}\left(\bar{\kappa }\right)}-\frac{1}{2}\right]\right\}, \end{array}$$

$$\begin{array}{c} k_{3}=\left\{\frac{I_{1}\left(\bar{\kappa }\right)}{I_{0}\left(\bar{\kappa }\right)}\left[\frac{{\bar{\kappa }}^{2}\delta^{N-1}}{\alpha \xi \left(n-1\right)}\left(\frac{1}{{\left(1+\xi \right)}^{N-1}}-1\right)-1\right]+\frac{\bar{\kappa }}{2}\right\}, \end{array}$$

$$\begin{array}{c} k_{4}=\frac{1+\left[\left(3-2N\right){\left(\xi \right)}^{2-N}+\left(2-N\right){\left(\xi \right)}^{3-N}-N{\left(\xi \right)}^{1-N}-{\left(\xi \right)}^{-N}\right]{\left(\frac{\xi +1}{\xi }\right)}^{-N}}{\left(N-2\right)\left(N-3\right)\xi^{2}}, \end{array}$$

$$k_{5}=\frac{\Gamma_{\alpha }\left\{\left[\left(n-1\right)\xi^{3}+\left(3n-3\right)\xi^{2}-2\xi -2n\right]{\left(\frac{\delta }{\xi +1}\right)}^{N}+2n\delta^{N}\right\}}{\left(6n^{2}-8n+2\right)\xi^{2}},$$

$$\begin{array}{c} k_{6}=k_{8}\left[-\frac{8}{3}\delta^{N}n^{2}\left(-\frac{5}{4}\xi n+\frac{1}{4}\xi +\Lambda \alpha \right)+\left(\xi +1\right){\left(\frac{\delta }{\xi +1}\right)}^{N}k_{9}\right], \end{array}$$

$$\begin{array}{c} k_{7}=\frac{I_{1}\left(\bar{\kappa }\right)}{I_{0}\left(\bar{\kappa }\right)}\bar{\kappa }\frac{\left[\left(n-1\right)\xi^{3}+\left(3n-3\right)\xi^{2}-2\xi -2n\right]{\left(\frac{\delta }{\xi +1}\right)}^{N}+2n\delta^{N}}{\delta \alpha \xi^{2}\left(n-1\right)\left(3n-1\right)}, \end{array}$$

$$k_{8}=\frac{3N}{4\alpha \delta \xi^{3}\left(15n^{2}-8n+1\right)\left(n-1\right)},$$

(A9) $$\begin{array}{c} k_{9}=\frac{5}{3}\xi n^{3}\left(\xi^{2}+2\xi -2\right)+k_{10}n^{2}+\frac{1}{3}\Lambda \xi^{2}\alpha {\left(\xi +2\right)}^{2}\\ -\frac{4}{3}\xi n\left(\xi +2\right)\left[\alpha \Lambda \left(\xi^{2}+2\xi -1\right)-\frac{1}{4}\xi \right], \end{array}$$

(A10) $$\begin{array}{c} k_{10}=\alpha \Lambda \left(\xi^{4}+4\xi^{3}+\frac{8}{3}\xi^{2}-\frac{8}{3}\xi +\frac{8}{3}\right)-2\xi^{3}-4\xi^{2}+\frac{2}{3}\xi , \end{array}$$

## Nomenclature

Symbol definition

*a*: sensitivity constant for the consistency index, K^−1^
*B_μ_*: sensitivity constant for the viscosity, K
*B_σ_*: sensitivity constant for the electrical conductivity, K^−1^
*C_p_*: specific heat, J·kg^−1^·K^−1^
*e*: electron charge, C
*E_0_*: external electric field, V·m^−1^
*E_z_*: electric field along the microcapillary, V·m^−1^
*h_∞_*: external convective heat transfer coefficient, W·m^−2^·K
*h_eq_*: equivalent heat transfer coefficient, W·m^−2^·K
*k*: thermal conductivity, W·m^−1^·K^−1^
*k_B_*: Boltzmann constant, J·K^−1^
*k_r_*: dimensionless ratio of thermal conductivities, *k_r_* = *k_i_*/*k_s_*
*k_T_*: equivalent Biot number, *k_T_* = *h_eq_t*/*k_s_*
*L*: microcapillary length, m
*m*: flow consistency index, Pa·s^*n*^
*n*: power-law index
*n_∞_*: bulk concentration of ions, m^−3^
*p*: pressure, kg·m^−1^·s^−2^
$\bar{p}$: dimensionless pressure
*p_e_*: Péclet numbers
*r, z*: radial and axial coordinates
*R*_1_: radius of the inner fluid, m
*R*_2_: external radius of the surrounding fluid, m
*R*_3_: external radius of the microcapillary wall, m
*R_r_*: ratio of radii, *R_r_* = *R*_2_/*R*_1_
*t*: thickness of the surrounding liquid, *t* = *R*_2_ − *R*_1_, m
*t_w_*: thickness of the microcapillary wall, *t_w_* = *R*_3_ − *R*_2_, m
*T*: temperature, K
*Q*: volumetric flow rate, m^3^·s^−1^
$\bar{Q}$: dimensionless volumetric flow rate
$\bar{u}$: dimensionless axial velocity
$\langle \bar{u}\rangle$: average dimensionless velocity
*u_c_*: Helmholtz-Smoluchowski velocity, m^3^·s^−1^
$\bar{v}$: dimensionless velocity component in radial direction
*v_r_*: velocity component in radial direction, m^3^·s^−1^
*v_z_*: fluid axial velocity, m^3^·s^−1^
*z*: valence

## Greek Letters

α: thermal diffusivity, $m^{2}$·$s^{-1}$; parameter, $\alpha =\mu_{r}u_{c}^{n-1}R_{1}/t^{n}$
$\bar{\alpha }$: conjugate heat transfer parameter, $\bar{\alpha }=\left(k_{i}/k_{s}\right)\left(R_{2}-R_{1}\right)/R_{1}$
$\mu_{r}$: dimensionless parameter, $\mu_{r}=m_{0}/\mu_{0}$
$\beta_{i}$: dimensionless parameter, $\beta_{i}=R_{1}/L$
$\beta_{s}$: dimensionless parameter, $\beta_{s}=t/L$
$\gamma_{a}$: dimensionless parameter, $\gamma_{a}=a▵T_{c}$
$\gamma_{\mu }$: dimensionless parameter, $\gamma_{\mu }=B_{\mu }▵T_{c}/T_{0}^{2}$
$\gamma_{\sigma }$: dimensionless parameter, $\gamma_{\sigma }=B_{\sigma }▵T_{c}$
$\gamma_{T}$: surface tension, N·$m^{-1}$
δ: dimensionless parameter, $\delta =2\bar{\kappa }I_{1}\left(\bar{\kappa }\right)/\alpha I_{0}\left(\bar{\kappa }\right)$
$\Gamma_{a}$: parameter, $\Gamma_{a}=aT_{0}^{2}/B_{\mu }$
$\Gamma_{\sigma }$: parameter, $\Gamma_{\sigma }=B_{\sigma }T_{0}^{{}^{2}}/B_{\mu }$
ϵ: dielectric permittivity, C·$V^{-1}$·$m^{-1}$
ζ: zeta potential, V
*Z*: dimensionless radial coordinate referred to the surrounding fluid
η: dimensionless radial coordinate referred to the inner fluid
θ: dimensionless temperature
κ: inverse Debye length, $m^{-1}$
$\kappa^{-1}$: Debye length, m
$\bar{\kappa }$: dimensionless parameter, $\bar{\kappa }=\kappa R_{1}$
λ: dimensionless temperature gradient, $\lambda ={\left.\left(\partial \theta_{i}/\partial \eta \right)\right|}_{\eta =1}$
Λ: dimensionless parameter, $\Lambda =t^{n+1}u_{c}^{1-n}/R_{1}^{2}\mu_{r}$
μ: viscosity of the conducting fluid, kg $m^{-1}$ $s^{-1}$
ξ: dimensionless parameter, $\xi =t/R_{1}$
ρ: density, kg·$m^{-3}$
$\rho_{e}$: net charge density, C·$m^{-3}$
σ: electrical conductivity, S·$m^{-1}$
$\sigma_{s}$: surface charge density at the interface, C·$m^{-2}$
$\tau_{rz}$: shear stress, N·$m^{-2}$
ϕ: external electric potential, V
$\bar{\phi }$: dimensionless external electric potential
χ: dimensionless axial coordinate
ψ: electric potential within the Debye length, V

## Subscripts

*c*: characteristic
*i*: inner fluid
*s*: surrounding fluid
*w*: wall

## Superscripts

(0): leading order
(1): first order
